# Crystal structure and Hirshfeld surface analysis of ethyl (3*E*)-5-(4-fluoro­phen­yl)3-{[(4-meth­oxy­phen­yl)formamido]­imino}-7-methyl-2*H*,3*H*,5*H*-[1,3]thia­zolo[3,2-*a*]pyrimidine-6-carboxyl­ate 0.25-hydrate

**DOI:** 10.1107/S2056989022006041

**Published:** 2022-08-09

**Authors:** Shaaban K. Mohamed, Joel T. Mague, Mehmet Akkurt, Abdallah M. Alfayomy, Fatma A. F. Ragab, Mokhtar A. Abd ul-Malik

**Affiliations:** a Chemistry and Environmental Division, Manchester Metropolitan University, Manchester, M1 5GD, England; b Chemistry Department, Faculty of Science, Minia University, 61519 El-Minia, Egypt; c Department of Chemistry, Tulane University, New Orleans, LA 70118, USA; d Department of Physics, Faculty of Sciences, Erciyes University, 38039 Kayseri, Turkey; e Department of Pharmaceutical Chemistry, Faculty of Pharmacy, Al-Azhar University, Assiut, 71524, Egypt; f Department of Pharmaceutical Chemistry, Faculty of Pharmacy, Cairo University, Kasr El-Aini Street, Cairo, PO Box, 11562, Egypt; g Chemistry Department, Faculty of Applied Science, Taiz University, Taiz, Yemen; Vienna University of Technology, Austria

**Keywords:** crystal structure, pyrimidine, thia­zole, hydrogen bond, Hirshfeld surface analysis

## Abstract

In the title compound, C_24_H_23_FN_4_O_4_S·0.25H_2_O, the di­hydro­pyrimidine ring is distinctly non-planar, with the flap C atom deviating by 0.297 (2) Å from the least-squares plane. In the crystal, zigzag chains are formed by N—H⋯N hydrogen bonds parallel to [010] and are connected into layers parallel to (100) by O—H⋯O, O—H⋯F, C—H⋯O, C—H⋯F and C—H⋯N hydrogen bonds. Additional C—H⋯O hydrogen bonds connect the layers into a three-dimensional network. A Hirshfeld surface analysis indicates that the most significant contributions to the crystal packing are from H⋯H (42.6%), O⋯H/H⋯O (16.8%) and C⋯H/H⋯C (15.5%) contacts.

## Abstract 2

The di­hydro­pyrimidine ring in the title mol­ecule is distinctly non-planar. In the crystal, zigzag chains parallel to [010] are formed by N—H⋯N hydrogen bonds and are connected into layers parallel to (100) by O—H⋯O, O—H⋯F, C—H⋯O, C—H⋯F and C—H⋯N hydrogen bonds. Further C—H⋯O hydrogen bonds connect the layers.

## 1. Chemical context

Inter­est in the anti­cancer activities of di­hydro­pyrimidines (DHPMs) has been increasing since 1999, when monastrol was discovered (Mayer *et al.*, 1999 ▸; Leizerman *et al.*, 2004 ▸). In addition, 1,3,4-oxa­diazole has been reported to exhibit a significant anti­cancer activity (Yadagiri *et al.*, 2015 ▸; Valente *et al.*, 2014 ▸; El-Din *et al.*, 2015 ▸). Since the combination of two or more pharmacophoric structural moieties can possibly augment the bioactivity, it was of inter­est to hybridize the DHPM moiety with 1,3,4-oxa­diazole, hoping to discover potent anti­cancer agents. 
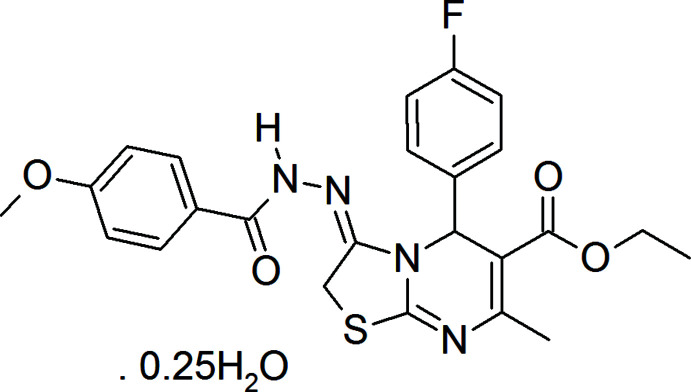


In this context, a target compound was designed through the condensation of 6-methyl-4-aryl-1,2,3,4-tetra­hydro­pyrim­idine-2(1*H*)-thione derivatives and 2-(chloro­meth­yl)-5-aryl-1,3,4-oxa­diazole derivatives (Ragab *et al.*, 2017 ▸). Unexpectedly, an intra­molecular cyclization and ring opening of 1,3,4-oxa­diazole occurred. The resulting product was chosen as an example of this series for further structural elucidation through X-ray crystallography. Herein we report the crystal structure and Hirshfeld analysis of the title compound, C_24_H_23_FN_4_O_4_S·0.25H_2_O.

## 2. Structural commentary

**Figure 1:**
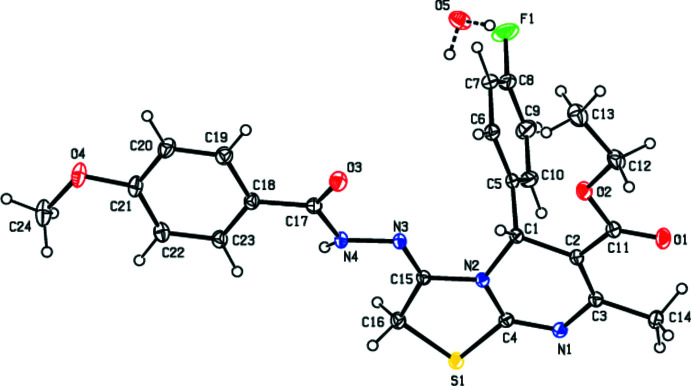
The title mol­ecule with the labelling scheme and displacement ellipsoids drawn at the 30% probability level.

In the title compound (Fig. 1 ▸), the di­hydro­pyrimidine portion (N1/C3/C2/C1/N2/C4) of the central ring is planar to within 0.0286 (9) Å (r.m.s. deviation of the fitted atoms = 0.0211 Å), with the flap C1 atom being 0.297 (2) Å out of this plane towards the bonded 4-fluoro­phenyl group. A puckering analysis (Cremer & Pople, 1975 ▸) of this ring yielded the parameters *Q* = 0.2074 (15) Å, θ = 112.1 (4)° and φ = 3.5 (4)°. The dihedral angle between the C5–C10 phenyl ring and the least-squares plane of the di­hydro­pyrimidine plane is 88.76 (5)°. The C4/N2/C15/C16/S1 ring is planar to within 0.0191 (8) Å (r.m.s. deviation of the fitted atoms = 0.0140 Å) and is inclined to the N1/C3/C2/C1/N2/C4 plane by 3.99 (9)°. The dihedral angle between the C4/N2/C15/C16/S1 ring and the C18–C23 phenyl ring is 9.28 (8)°.

## 3. Supra­molecular features

**Figure 2:**
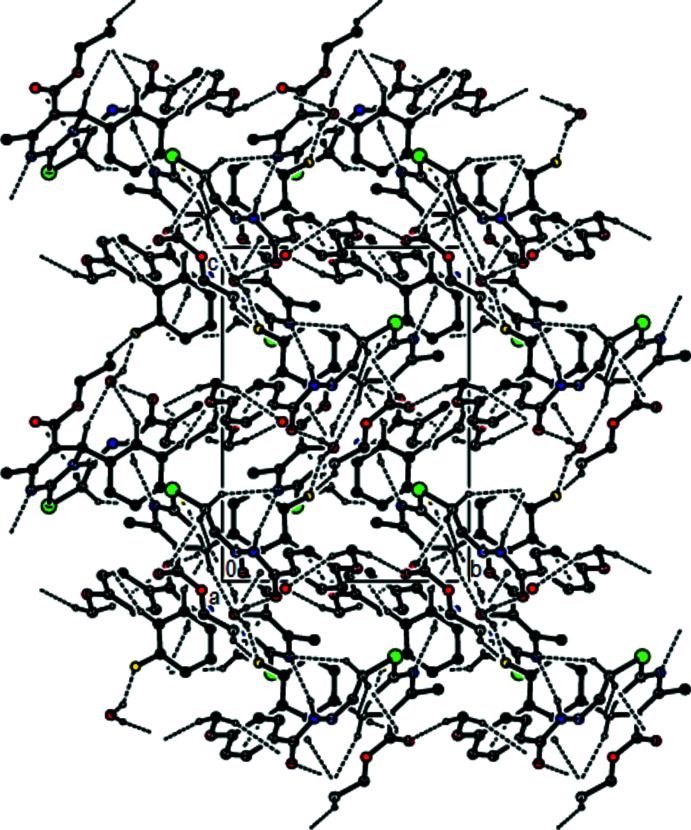
View of the mol­ecular packing along [100]. O—H⋯O, O—H⋯F, C—H⋯ O, C—H⋯N and C—H⋯F hydrogen bonds are shown as dashed lines.

In the crystal, mol­ecules are connected into zigzag chains running parallel to [010] by N4—H4⋯N1 hydrogen bonds (Table 1 ▸). The chains are connected into (100) layers by O5—H5*B*⋯O3 and O5—H5*A*⋯F1 hydrogen bonds involving the water mol­ecule, as well as by C13—H13*B*⋯F1, C16—H16*A*⋯N1 and all of the C—H⋯O hydrogen bonds listed in Table 1 ▸, except for the C24—H24*C*⋯O1 hydrogen bond (Figs. 2 ▸, 3 ▸ and 4 ▸) that serves to link the layers into a three-dimensional network.

## 4. Hirshfeld surface analysis

**Figure 5:**
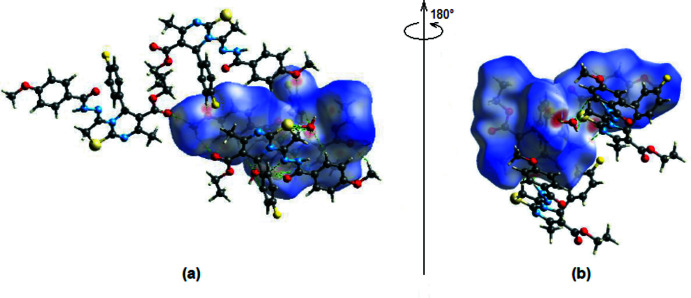
(*a*) Front and (*b*) back sides of the three-dimensional Hirshfeld surface of the title compound mapped over *d*_norm_, with a fixed colour scale of −0.7253 (red) to +1.4745 (blue) a.u.

A Hirshfeld surface analysis was performed, and two-dimensional fingerprint plots were constructed using *Crystal Explorer17.5* to qu­antify the inter­molecular inter­actions in the title mol­ecule (Turner *et al.*, 2017 ▸). Fig. 5 ▸ depicts the Hirshfeld surface plotted over *d*_norm_ in the range −0.7253 to +1.4745 arbitrary units, with red patches indicating putative hydrogen bonding in the crystal structure.

**Figure 6:**
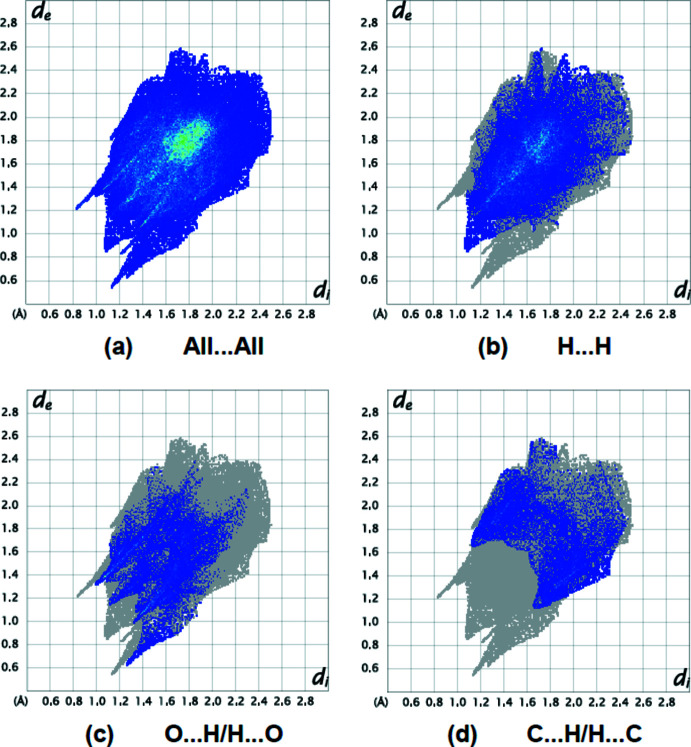
Two-dimensional fingerprint plots for the title compound, showing (*a*) all inter­actions, and delineated into (*b*) H⋯H, (*c*) O⋯H/H⋯O and (*d*) C⋯H/H⋯C inter­actions. The *d*_i_ and *d*_e_ values are the closest inter­nal and external distances (in Å) from given points on the Hirshfeld surface.

The intensity of the red patches is more pronounced for N4—H4⋯N1, C1—H1⋯O5, C16—H16*B*⋯O5, C24—H24*A*⋯O1, C24—H24*C*⋯O1 and O5—H5*B*⋯O3, thus revealing the strongest inter­actions when compared to other red spots on the Hirshfeld surface. Table 2 ▸ gives numerical data for close inter­molecular contacts. The two-dimensional fingerprint plots (Fig. 6 ▸) shows that the largest contributions are from H⋯H (42.6%; Fig. 6 ▸ *b*), O⋯H/H⋯O (16.8%; Fig. 6 ▸ *c*) and C⋯H/H⋯C (15.5%; Fig. 6 ▸ *d*) inter­actions. Other inter­actions contributing less to the crystal packing are from F⋯H/H⋯F (6.7%), N⋯H/H⋯N (4.5%), S⋯H/H⋯S (3.4%), S⋯C/C⋯S (3.4%), C⋯C (2.8%), S⋯N/N⋯S (1.4%),N⋯C/C⋯N (1.4%), O⋯C/C⋯O (0.7%), N⋯N (0.5%), O⋯N/N⋯O (0.2%) and S⋯O/O⋯S (0.1%) inter­actions.

## 5. Database survey

A search of the Cambridge Structural Database (CSD, Version 5.42, update of September 2021; Groom *et al.*, 2016 ▸) for compounds most closely related to the 2,3-di­hydro-5*H*-[1,3]thia­zolo[3,2-*a*]pyrimidine unit of the title compound gave the following hits: refcodes ZOWXAM (**I**) (Krishnamurthy *et al.*, 2014 ▸); PONVOF (**II**) (Krishnamurthy & Begum, 2014 ▸); AFIZUM (**III**) (Fathima *et al.*, 2013 ▸); YAYHAJ (**IV**) (Nagarajaiah *et al.*, 2012 ▸); KUSQUL (**V**) (Jotani *et al.*, 2010*a* ▸); PUJRIW (**VI**) (Jotani *et al.*, 2010*b* ▸); DIWSIM (**VII**) (Jotani & Baldaniya, 2008 ▸); TICHAP (**VIII**) (Jotani & Baldaniya, 2007 ▸); AWUPAK (**IX**) (Fun *et al.*, 2011 ▸); XETKOX (**X**) (Sridhar *et al.*, 2006 ▸) and XETKOX01 (**XI**) (Sridhar *et al.*, 2006 ▸).

In the crystal of (**I**), pairs of weak C—H⋯O hydrogen bonds link mol­ecules related by twofold rotation axes, forming 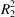 (10) rings, which in turn are linked by weak C—H⋯N inter­actions to form chains parallel to [010]. In addition, weak C—H⋯π(arene) inter­actions link the chains into layers parallel to (001), and π–π inter­actions connect these layers into a three-dimensional network.

In (**II**), weak C—H⋯F and C— H⋯O hydrogen bonds connect mol­ecules, forming zigzag chains parallel to [010]. In addition, π–π stacking inter­actions connect these chains into ladders *via* inversion-related 4-fluoro­phenyl groups.

In (**III**), pairs of weak C—H⋯O hydrogen bonds lead to the formation of inversion dimers. A weak C—H⋯π inter­action and π–π stacking inter­actions are observed.

In (**IV**), O—H⋯N and C— H⋯S inter­actions result in (001) layers. The supra­molecular assembly is stabilized by π–π stacking inter­actions between the 2-bromo­benzyl­idene and thia­zolo­pyrimidine rings. In addition, C—H⋯π inter­actions are also observed.

In (**V**), co-operative C—H⋯O and C—H⋯π inter­actions lead to supra­molecular chains parallel [100]. These chains are connected *via* π–π inter­actions.

The crystal packing of (**VI**) is influenced by weak inter­molecular C—H⋯π inter­actions and π–π stacking between the thia­zole and phenyl rings, which stack the mol­ecules parallel to [001].

In (**VII**), in addition to inter­molecular C— H⋯O hydrogen bonding, short intra­molecular C—H⋯S contacts and π–π stacking inter­actions contribute to the crystal packing.

In (**VIII**), short inter­molecular C—H⋯O, C—H⋯π and π–π stacking inter­actions contribute to the stability of the crystal packing.

In (**IX**), mol­ecules are linked into a three-dimensional network by inter­molecular C— H⋯O and C—H⋯F hydrogen bonds. The crystal structure is further stabilized by a C—H⋯π inter­action.

Compounds (**X**) and (**XI**) crystallize in two polymorphic forms having the same space-group type, *viz. P*1, with *Z*′ = 2 and *Z*′ = 1. In both polymorphs, the mol­ecules are linked by N—H⋯O and C—H⋯O hydrogen bonds.

## 6. Synthesis and crystallization

A mixture of ethyl 4-(4-fluoro­phen­yl)-6-methyl-2-thioxo-1,2,3,4-tetra­hydro­pyrimidine-5-carboxyl­ate (2 mmol), 2-(chlor­o­meth­yl)-5-(4-meth­oxy­phen­yl)-1,3,4-oxa­diazole (2 mmol), potassium iodide (2 mmol) and triethyl amine (2.5 mmol), was refluxed for 4 h in absolute ethanol (20 ml). The reaction mixture was poured onto crushed ice (40 g) and acidified with acetic acid (2 ml). The deposited precipitate was filtered off, washed with cold water, dried and recrystallized from a methanol/DMF mixture.

Yield: 95%; melting point: 493–495 K; IR (KBr) ν_max_/cm^−1^ 3390, 3178, 1693, 1654. ^1^H NMR (400 MHz, DMSO-*d*_6_) δ 10.60 (*s*, 1H, NH), 7.81 (*d*, *J* = 8.7 Hz, 2H, Ar—H), 7.44 (*t*, *J* = 7.7 Hz, 2H, Ar—H), 7.15 (*t*, *J* = 7.7 Hz, 2H, Ar—H), 7.03 (*d*, *J* = 8.7 Hz, 2H, Ar—H), 6.13 (*s*, 1H, C4—H), 4.45 (*d*, *J* = 17.4 Hz, 1H, S—CH_2_), 4.35 (*d*, *J* = 17.3 Hz, 1H, S—CH_2_), 4.03 (*q*, *J* = 7.1 Hz, 2H, CH_2_—CH_3_), 3.82 (*s*, 3H, OCH_3_), 2.34 (*s*, 3H, C6-CH_3_), 1.11 (*t*, *J* = 7.1 Hz, 3H, CH_2_—CH_3_). ^13^C NMR (125 MHz, DMSO-*d*_6_) δ 165.59, 163.23, 163.20, 162.65, 153.92, 153.58, 130.57, 130.50, 130.03, 125.90, 115.64, 115.47, 114.05, 105.95, 60.28, 55.87, 54.89, 28.56, 23.06, 14.45. Analysis calculated for C_24_H_23_FN_4_O_4_S (482.53): C 59.74, H 4.80, N 11.61. Found: C 60.02, H 4.89, N 11.87.

## 7. Refinement

Crystal data, data collection and structure refinement details are summarized in Table 3 ▸. The H atoms were found in difference-Fourier maps; all C and N-bound H atoms were refined freely. The water mol­ecule was found to be occupationally disordered and was refined with a fixed site occupation factor of 1/4. The H atoms of the water mol­ecules were located in a difference-Fourier map, their bond lengths set to an ideal value of 0.87 Å, and were refined with *U*_iso_(H) = 1.5 *U*_eq_(O) using a riding model.

## Supplementary Material

Crystal structure: contains datablock(s) I, global. DOI: 10.1107/S2056989022006041/wm5648sup1.cif

Structure factors: contains datablock(s) I. DOI: 10.1107/S2056989022006041/wm5648Isup2.hkl

Supporting information file. DOI: 10.1107/S2056989022006041/wm5648Isup3.cml

CCDC reference: 2177565

Additional supporting information:  crystallographic information; 3D view; checkCIF report

## Figures and Tables

**Figure 3:**
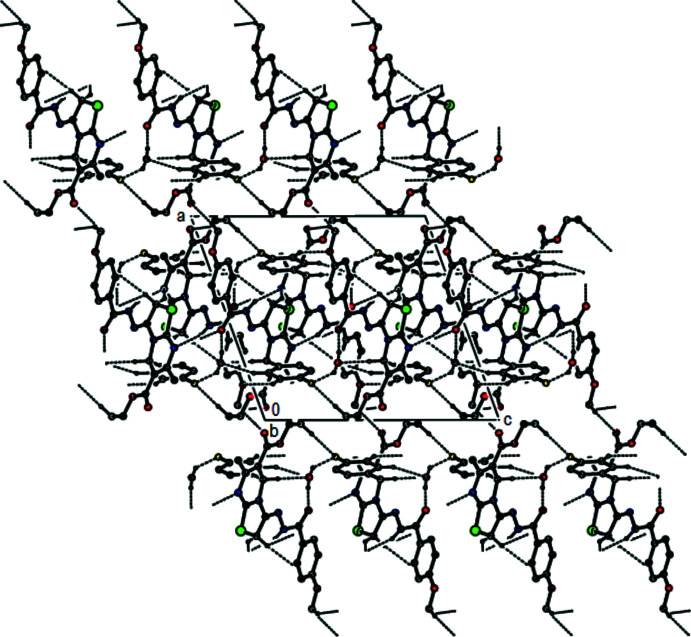
View of the mol­ecular packing along [010]. Hydrogen bonds are depicted as in Fig. 2 ▸.

**Figure 4:**
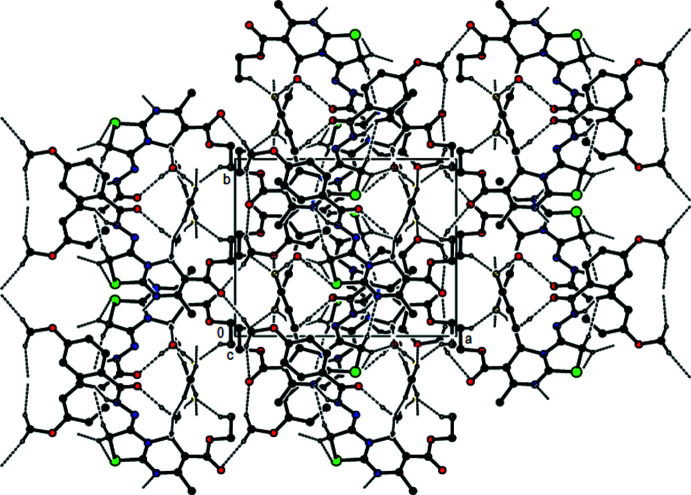
View of the mol­ecular packing along [001]. Hydrogen bonds are depicted as in Fig. 2 ▸.

**Table 1:** Hydrogen-bond geometry (Å, °)

*D*—H⋯*A*	*D*—H	H⋯*A*	*D*⋯*A*	*D*—H⋯*A*
N4—H4⋯N1^i^	0.885 (19)	2.164 (19)	2.9888 (17)	154.8 (16)
C1—H1⋯O5^ii^	0.987 (17)	2.345 (18)	3.307 (5)	164.7 (13)
C7—H7⋯O4^iii^	0.97 (2)	2.41 (2)	3.285 (2)	148.7 (17)
C13—H13*B*⋯F1^ii^	0.98 (3)	2.49 (3)	3.386 (2)	153.0 (19)
C16—H16*A*⋯N1^i^	0.98 (2)	2.58 (2)	3.4019 (19)	142.4 (15)
C16—H16*B*⋯O5^iv^	0.98 (2)	2.37 (2)	3.282 (5)	154.6 (16)
C24—H24*A*⋯O1^v^	0.99 (2)	2.53 (2)	3.450 (3)	154.6 (18)
C24—H24*C*⋯O1^vi^	0.95 (2)	2.57 (2)	3.504 (2)	167.8 (17)
O5—H5*A*⋯F1	0.87	1.76	2.479 (5)	138
O5—H5*B*⋯O3^vii^	0.87	2.00	2.863 (5)	174

Symmetry codes: (i) 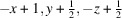 ; (ii) 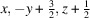 ; (iii) 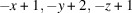 ; (iv) 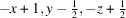 ; (v) 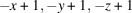 ; (vi) 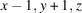 ; (vii) 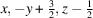 .

**Table 2:** Summary of short inter­atomic contacts (Å) in the title compound. Asterisks relate to atoms of the underoccupied water mol­ecule.

Contact	Distance	Symmetry operation
N1⋯H4	2.165	1 − *x*, − 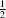 + *y*, 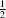 − *z*
F1⋯H13*B*	2.49	*x*, 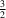 − *y*, − 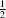 + *z*
F1⋯*H5*A*	1.76	*x*, *y*, *z*
F1⋯H14*C*	2.66	*x*, 1 + *y*, *z*
H14*A*⋯H24*C*	2.56	1 + *x*, −1 + *y*, *z*
H12*C*⋯O1	2.66	2 − *x*, 1 − *y*, 1 − *z*
H16*B*⋯O3	2.49	1 − *x*, 1 − *y*, 1 − *z*
O3⋯*H5*B*	2.00	*x*, 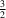 − *y*, 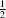 + *z*
H7⋯O4	2.41	1 − *x*, 2 − *y*, 1 − *z*
H16*B*⋯*H5*B*	2.05	1 − *x*, − 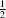 + *y*, 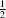 − *z*
H13*A*⋯H24*B*	2.41	1 + *x*, 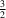 − *y*, 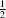 + *z*
H14*C*⋯*O5	2.87	*x*, −1 + *y*, *z*

**Table 3:** Experimental details

Crystal data	
Chemical formula	C_24_H_23_FN_4_O_4_S·0.25H_2_O
*M*_r_	487.03
Crystal system, space group	Monoclinic, *P*2_1_/*c*
Temperature (K)	150
*a*, *b*, *c* (Å)	14.4316 (3), 10.8518 (2), 15.5940 (3)
β (°)	109.941 (1)
*V* (Å^3^)	2295.74 (8)
*Z*	4
Radiation type	Cu *K*α
μ (mm^−1^)	1.68
Crystal size (mm)	0.15 × 0.14 × 0.11

Data collection	
Diffractometer	Bruker D8 VENTURE PHOTON 100 CMOS
Absorption correction	Multi-scan (*SADABS*; Krause *et al.*, 2015 ▸)
*T*_min_, *T*_max_	0.75, 0.84
No. of measured, independent and observed [*I* > 2σ(*I*)] reflections	17597, 4576, 4142
*R*_int_	0.029
(sin θ/λ)_max_ (Å^−1^)	0.625

Refinement	
*R*[*F* ^2^ > 2σ(*F* ^2^)], *wR*(*F* ^2^), *S*	0.035, 0.088, 1.04
No. of reflections	4576
No. of parameters	409
H-atom treatment	H atoms treated by a mixture of independent and constrained refinement
Δρ_max_, Δρ_min_ (e Å^−3^)	0.34, −0.56

Computer programs: *APEX3* and *SAINT* (Bruker, 2016 ▸), *SHELXT* (Sheldrick, 2015*a* ▸), *SHELXL* (Sheldrick, 2015*b* ▸), *DIAMOND* (Brandenburg & Putz, 2012 ▸) and *publCIF* (Westrip, 2010 ▸).

## supplementary crystallographic information

### Crystal data

**Table:**

C_24_H_23_FN_4_O_4_S·0.25H_2_O	*F*(000) = 1018
*M**_r_* = 487.03	*D*_x_ = 1.409 Mg m^−^^3^
Monoclinic, *P*2_1_/*c*	Cu *K*α radiation, λ = 1.54178 Å
*a* = 14.4316 (3) Å	Cell parameters from 9970 reflections
*b* = 10.8518 (2) Å	θ = 3.3–74.4°
*c* = 15.5940 (3) Å	µ = 1.68 mm^−^^1^
β = 109.941 (1)°	*T* = 150 K
*V* = 2295.74 (8) Å^3^	Block, colourless
*Z* = 4	0.15 × 0.14 × 0.11 mm

### Data collection

**Table:**

Bruker D8 VENTURE PHOTON 100 CMOS diffractometer	4576 independent reflections
Radiation source: INCOATEC IµS micro–focus source	4142 reflections with *I* > 2σ(*I*)
Mirror monochromator	*R*_int_ = 0.029
Detector resolution: 10.4167 pixels mm^-1^	θ_max_ = 74.4°, θ_min_ = 3.3°
ω scans	*h* = −17→18
Absorption correction: multi-scan (*SADABS*; Krause *et al.*, 2015)	*k* = −13→12
*T*_min_ = 0.75, *T*_max_ = 0.84	*l* = −19→18
17597 measured reflections	

### Refinement

**Table:**

Refinement on *F*^2^	Secondary atom site location: difference Fourier map
Least-squares matrix: full	Hydrogen site location: difference Fourier map
*R*[*F*^2^ > 2σ(*F*^2^)] = 0.035	H atoms treated by a mixture of independent and constrained refinement
*wR*(*F*^2^) = 0.088	*w* = 1/[σ^2^(*F*_o_^2^) + (0.0389*P*)^2^ + 1.1637*P*] where *P* = (*F*_o_^2^ + 2*F*_c_^2^)/3
*S* = 1.04	(Δ/σ)_max_ < 0.001
4576 reflections	Δρ_max_ = 0.34 e Å^−^^3^
409 parameters	Δρ_min_ = −0.56 e Å^−^^3^
0 restraints	Extinction correction: *SHELXL-2018/1* (Sheldrick, 2015*b*), Fc^*^=kFc[1+0.001xFc^2^λ^3^/sin(2θ)]^-1/4^
Primary atom site location: structure-invariant direct methods	Extinction coefficient: 0.00229 (19)

### Special details

**Table:**

Geometry. All esds (except the esd in the dihedral angle between two l.s. planes) are estimated using the full covariance matrix. The cell esds are taken into account individually in the estimation of esds in distances, angles and torsion angles; correlations between esds in cell parameters are only used when they are defined by crystal symmetry. An approximate (isotropic) treatment of cell esds is used for estimating esds involving l.s. planes.
Refinement. Refinement of F^2^ against ALL reflections. The weighted R-factor wR and goodness of fit S are based on F^2^, conventional R-factors R are based on F, with F set to zero for negative F^2^. The threshold expression of F^2^ > 2sigma(F^2^) is used only for calculating R-factors(gt) etc. and is not relevant to the choice of reflections for refinement. R-factors based on F^2^ are statistically about twice as large as those based on F, and R- factors based on ALL data will be even larger. Refinement of the site occupancy factor for the lattice water (O5) converged at *ca.* 0.25. This was fixed at this value for the remainder of the refinement, the attached hydrogen atoms were located in a difference map and included as riding contributions in idealized positions.

### Fractional atomic coordinates and isotropic or equivalent isotropic displacement parameters (Å^2^)

**Table:**

	*x*	*y*	*z*	*U*_iso_*/*U*_eq_	Occ. (<1)
S1	0.45709 (2)	0.29846 (3)	0.22568 (2)	0.02637 (11)	
F1	0.81951 (11)	0.85095 (10)	0.24433 (11)	0.0709 (4)	
O1	0.93592 (8)	0.24379 (11)	0.47803 (8)	0.0365 (3)	
O2	0.88439 (7)	0.41670 (10)	0.52684 (7)	0.0294 (2)	
O3	0.55706 (8)	0.71812 (10)	0.54550 (7)	0.0307 (2)	
O4	0.17420 (9)	1.04606 (11)	0.46165 (8)	0.0390 (3)	
N1	0.64445 (8)	0.22708 (11)	0.27415 (8)	0.0220 (2)	
N2	0.60560 (8)	0.39869 (10)	0.34918 (8)	0.0200 (2)	
N3	0.54560 (8)	0.55654 (10)	0.41266 (8)	0.0206 (2)	
N4	0.46512 (8)	0.62737 (11)	0.41154 (8)	0.0209 (2)	
H4	0.4182 (14)	0.6419 (17)	0.3587 (13)	0.032 (5)*	
C1	0.70873 (9)	0.43284 (13)	0.40035 (10)	0.0213 (3)	
H1	0.7122 (12)	0.4512 (15)	0.4634 (11)	0.022 (4)*	
C2	0.77324 (10)	0.32264 (13)	0.39918 (10)	0.0224 (3)	
C3	0.74171 (10)	0.23233 (13)	0.33574 (10)	0.0224 (3)	
C4	0.58287 (10)	0.30597 (12)	0.28731 (9)	0.0207 (3)	
C5	0.73684 (9)	0.54708 (13)	0.35807 (10)	0.0233 (3)	
C6	0.76870 (10)	0.65331 (14)	0.40974 (11)	0.0291 (3)	
H6	0.7696 (14)	0.6545 (17)	0.4744 (13)	0.035 (5)*	
C7	0.79756 (12)	0.75620 (15)	0.37183 (14)	0.0365 (4)	
H7	0.8223 (15)	0.829 (2)	0.4092 (14)	0.048 (6)*	
C8	0.79149 (13)	0.75033 (15)	0.28209 (14)	0.0410 (4)	
C9	0.75793 (15)	0.64862 (16)	0.22793 (14)	0.0420 (4)	
H9	0.7526 (16)	0.651 (2)	0.1629 (15)	0.050 (6)*	
C10	0.73134 (12)	0.54590 (14)	0.26730 (11)	0.0314 (3)	
H10	0.7067 (14)	0.4745 (19)	0.2304 (13)	0.038 (5)*	
C11	0.87186 (10)	0.32007 (13)	0.46924 (10)	0.0253 (3)	
C12	0.97996 (11)	0.42561 (17)	0.59855 (11)	0.0331 (3)	
H12A	0.9878 (15)	0.353 (2)	0.6404 (14)	0.045 (5)*	
H12B	1.0316 (14)	0.4216 (17)	0.5703 (12)	0.034 (5)*	
H12C	0.9642 (16)	0.618 (2)	0.6014 (15)	0.052 (6)*	
C13	0.98133 (13)	0.5454 (2)	0.64668 (13)	0.0412 (4)	
H13A	1.0491 (17)	0.557 (2)	0.6929 (15)	0.055 (6)*	
H13B	0.9329 (18)	0.545 (2)	0.6776 (16)	0.060 (7)*	
C14	0.80354 (11)	0.12659 (15)	0.32406 (12)	0.0287 (3)	
H14A	0.8649 (17)	0.155 (2)	0.3186 (15)	0.052 (6)*	
H14B	0.8240 (16)	0.074 (2)	0.3764 (16)	0.054 (6)*	
H14C	0.7663 (16)	0.079 (2)	0.2696 (15)	0.046 (5)*	
C15	0.52713 (9)	0.47098 (12)	0.35314 (9)	0.0202 (3)	
C16	0.43099 (10)	0.43208 (14)	0.28344 (10)	0.0260 (3)	
H16A	0.4032 (14)	0.4974 (19)	0.2389 (13)	0.040 (5)*	
H16B	0.3839 (15)	0.4099 (18)	0.3141 (14)	0.043 (5)*	
C17	0.48023 (10)	0.71162 (13)	0.48039 (9)	0.0223 (3)	
C18	0.39469 (10)	0.79431 (13)	0.47152 (9)	0.0230 (3)	
C19	0.41359 (12)	0.90278 (14)	0.52329 (10)	0.0289 (3)	
H19	0.4793 (15)	0.9188 (17)	0.5629 (13)	0.036 (5)*	
C20	0.33869 (12)	0.98472 (15)	0.51747 (11)	0.0331 (3)	
H20	0.3508 (16)	1.062 (2)	0.5510 (15)	0.053 (6)*	
C21	0.24267 (11)	0.95909 (15)	0.46124 (10)	0.0294 (3)	
C22	0.22193 (11)	0.85134 (15)	0.40997 (10)	0.0287 (3)	
H22	0.1533 (15)	0.8314 (18)	0.3709 (13)	0.037 (5)*	
C23	0.29829 (11)	0.76979 (14)	0.41584 (10)	0.0260 (3)	
H23	0.2829 (13)	0.6946 (17)	0.3814 (12)	0.031 (5)*	
C24	0.07367 (14)	1.0253 (2)	0.40748 (14)	0.0456 (5)	
H24A	0.0502 (16)	0.946 (2)	0.4247 (15)	0.054 (6)*	
H24B	0.0650 (16)	1.025 (2)	0.3411 (16)	0.053 (6)*	
H24C	0.0390 (16)	1.093 (2)	0.4203 (14)	0.048 (6)*	
O5	0.7191 (3)	0.9508 (5)	0.0994 (3)	0.0378 (10)	0.25
H5A	0.766259	0.901704	0.130626	0.057*	0.25
H5B	0.667996	0.902764	0.079072	0.057*	0.25

### Atomic displacement parameters (Å^2^)

**Table:**

	*U*^11^	*U*^22^	*U*^33^	*U*^12^	*U*^13^	*U*^23^
S1	0.01757 (17)	0.0260 (2)	0.0312 (2)	0.00125 (12)	0.00266 (13)	−0.00720 (13)
F1	0.1061 (11)	0.0263 (6)	0.1191 (11)	−0.0171 (6)	0.0888 (10)	−0.0055 (6)
O1	0.0214 (5)	0.0345 (6)	0.0476 (7)	0.0093 (4)	0.0038 (5)	−0.0055 (5)
O2	0.0177 (5)	0.0313 (6)	0.0326 (6)	0.0026 (4)	0.0002 (4)	−0.0057 (4)
O3	0.0271 (5)	0.0336 (6)	0.0274 (5)	0.0038 (4)	0.0040 (4)	−0.0039 (4)
O4	0.0370 (6)	0.0405 (7)	0.0400 (6)	0.0175 (5)	0.0137 (5)	−0.0051 (5)
N1	0.0198 (5)	0.0194 (6)	0.0257 (6)	0.0010 (4)	0.0064 (5)	−0.0013 (4)
N2	0.0151 (5)	0.0186 (6)	0.0246 (6)	0.0011 (4)	0.0046 (4)	−0.0013 (4)
N3	0.0174 (5)	0.0202 (6)	0.0246 (6)	0.0028 (4)	0.0078 (4)	0.0011 (4)
N4	0.0179 (5)	0.0216 (6)	0.0231 (6)	0.0044 (4)	0.0069 (5)	0.0005 (4)
C1	0.0151 (6)	0.0211 (7)	0.0256 (7)	0.0001 (5)	0.0042 (5)	−0.0028 (5)
C2	0.0169 (6)	0.0208 (7)	0.0284 (7)	0.0019 (5)	0.0062 (5)	0.0005 (5)
C3	0.0190 (6)	0.0205 (7)	0.0276 (7)	0.0016 (5)	0.0079 (5)	0.0023 (5)
C4	0.0206 (6)	0.0181 (7)	0.0230 (7)	−0.0001 (5)	0.0067 (5)	0.0005 (5)
C5	0.0146 (6)	0.0199 (7)	0.0348 (8)	0.0012 (5)	0.0077 (5)	−0.0021 (5)
C6	0.0207 (7)	0.0237 (8)	0.0399 (9)	0.0007 (5)	0.0066 (6)	−0.0057 (6)
C7	0.0270 (8)	0.0204 (8)	0.0640 (11)	−0.0040 (6)	0.0179 (8)	−0.0093 (7)
C8	0.0439 (9)	0.0204 (8)	0.0744 (13)	−0.0032 (7)	0.0406 (9)	0.0001 (8)
C9	0.0582 (11)	0.0270 (9)	0.0568 (11)	−0.0018 (7)	0.0402 (10)	−0.0014 (7)
C10	0.0373 (8)	0.0227 (8)	0.0403 (9)	−0.0035 (6)	0.0212 (7)	−0.0051 (6)
C11	0.0191 (6)	0.0238 (7)	0.0312 (7)	0.0015 (5)	0.0064 (6)	−0.0005 (6)
C12	0.0177 (7)	0.0431 (10)	0.0315 (8)	0.0004 (6)	−0.0005 (6)	−0.0031 (7)
C13	0.0284 (8)	0.0560 (12)	0.0348 (9)	−0.0025 (8)	0.0053 (7)	−0.0140 (8)
C14	0.0239 (7)	0.0252 (8)	0.0356 (8)	0.0050 (6)	0.0083 (6)	−0.0037 (6)
C15	0.0171 (6)	0.0186 (7)	0.0246 (7)	0.0011 (5)	0.0068 (5)	0.0028 (5)
C16	0.0187 (6)	0.0254 (8)	0.0303 (8)	0.0020 (5)	0.0038 (6)	−0.0048 (6)
C17	0.0226 (7)	0.0217 (7)	0.0234 (7)	0.0007 (5)	0.0090 (5)	0.0015 (5)
C18	0.0246 (7)	0.0235 (7)	0.0234 (7)	0.0023 (5)	0.0113 (6)	0.0002 (5)
C19	0.0287 (8)	0.0288 (8)	0.0292 (8)	0.0003 (6)	0.0098 (6)	−0.0039 (6)
C20	0.0378 (9)	0.0286 (8)	0.0336 (8)	0.0043 (6)	0.0134 (7)	−0.0072 (6)
C21	0.0322 (8)	0.0313 (8)	0.0286 (8)	0.0110 (6)	0.0155 (6)	0.0018 (6)
C22	0.0254 (7)	0.0341 (8)	0.0283 (7)	0.0042 (6)	0.0114 (6)	−0.0016 (6)
C23	0.0256 (7)	0.0274 (8)	0.0273 (7)	0.0014 (6)	0.0121 (6)	−0.0035 (6)
C24	0.0357 (9)	0.0540 (12)	0.0455 (11)	0.0212 (9)	0.0119 (8)	−0.0026 (9)
O5	0.027 (2)	0.042 (3)	0.039 (3)	−0.0069 (19)	0.0036 (19)	0.006 (2)

### Geometric parameters (Å, º)

**Table:**

S1—C4	1.7422 (14)	C9—C10	1.388 (2)
S1—C16	1.8130 (15)	C9—H9	0.99 (2)
F1—C8	1.3657 (19)	C10—H10	0.96 (2)
O1—C11	1.2133 (18)	C12—C13	1.498 (2)
O2—C11	1.3522 (18)	C12—H12A	1.00 (2)
O2—C12	1.4526 (17)	C12—H12B	0.988 (19)
O3—C17	1.2236 (17)	C13—H12C	1.03 (2)
O4—C21	1.3680 (18)	C13—H13A	1.00 (2)
O4—C24	1.426 (2)	C13—H13B	0.98 (3)
N1—C4	1.2996 (18)	C14—H14A	0.97 (2)
N1—C3	1.4058 (17)	C14—H14B	0.96 (2)
N2—C4	1.3546 (17)	C14—H14C	0.98 (2)
N2—C15	1.3963 (17)	C15—C16	1.5020 (19)
N2—C1	1.4762 (16)	C16—H16A	0.98 (2)
N3—C15	1.2750 (18)	C16—H16B	0.98 (2)
N3—N4	1.3880 (15)	C17—C18	1.4939 (19)
N4—C17	1.3698 (18)	C18—C23	1.391 (2)
N4—H4	0.885 (19)	C18—C19	1.401 (2)
C1—C2	1.5194 (18)	C19—C20	1.379 (2)
C1—C5	1.5226 (19)	C19—H19	0.96 (2)
C1—H1	0.987 (17)	C20—C21	1.392 (2)
C2—C3	1.356 (2)	C20—H20	0.97 (2)
C2—C11	1.4694 (19)	C21—C22	1.390 (2)
C3—C14	1.5024 (19)	C22—C23	1.392 (2)
C5—C10	1.391 (2)	C22—H22	0.99 (2)
C5—C6	1.392 (2)	C23—H23	0.960 (19)
C6—C7	1.392 (2)	C24—H24A	0.99 (2)
C6—H6	1.004 (19)	C24—H24B	1.00 (2)
C7—C8	1.373 (3)	C24—H24C	0.95 (2)
C7—H7	0.97 (2)	O5—H5A	0.8700
C8—C9	1.374 (3)	O5—H5B	0.8701
			
C4—S1—C16	92.44 (6)	C13—C12—H12B	112.3 (11)
C11—O2—C12	116.09 (11)	H12A—C12—H12B	108.7 (16)
C21—O4—C24	118.60 (14)	C12—C13—H12C	111.3 (12)
C4—N1—C3	116.27 (12)	C12—C13—H13A	107.9 (13)
C4—N2—C15	116.48 (11)	H12C—C13—H13A	110.2 (18)
C4—N2—C1	121.74 (11)	C12—C13—H13B	110.9 (14)
C15—N2—C1	121.23 (11)	H12C—C13—H13B	107.1 (18)
C15—N3—N4	115.33 (11)	H13A—C13—H13B	109.4 (19)
C17—N4—N3	116.80 (11)	C3—C14—H14A	111.4 (13)
C17—N4—H4	118.6 (12)	C3—C14—H14B	112.1 (14)
N3—N4—H4	118.8 (12)	H14A—C14—H14B	103.9 (18)
N2—C1—C2	107.75 (11)	C3—C14—H14C	109.4 (12)
N2—C1—C5	109.80 (11)	H14A—C14—H14C	109.8 (18)
C2—C1—C5	112.38 (11)	H14B—C14—H14C	110.1 (18)
N2—C1—H1	106.8 (9)	N3—C15—N2	118.03 (12)
C2—C1—H1	110.3 (9)	N3—C15—C16	130.06 (12)
C5—C1—H1	109.6 (10)	N2—C15—C16	111.90 (11)
C3—C2—C11	121.84 (12)	C15—C16—S1	106.69 (9)
C3—C2—C1	121.53 (12)	C15—C16—H16A	111.4 (12)
C11—C2—C1	116.62 (12)	S1—C16—H16A	109.4 (11)
C2—C3—N1	122.42 (12)	C15—C16—H16B	109.7 (12)
C2—C3—C14	125.10 (13)	S1—C16—H16B	109.9 (12)
N1—C3—C14	112.47 (12)	H16A—C16—H16B	109.8 (16)
N1—C4—N2	126.10 (12)	O3—C17—N4	123.09 (13)
N1—C4—S1	121.47 (10)	O3—C17—C18	121.90 (13)
N2—C4—S1	112.41 (10)	N4—C17—C18	115.00 (12)
C10—C5—C6	119.33 (14)	C23—C18—C19	118.49 (13)
C10—C5—C1	120.14 (13)	C23—C18—C17	124.17 (13)
C6—C5—C1	120.53 (13)	C19—C18—C17	117.34 (13)
C5—C6—C7	120.41 (16)	C20—C19—C18	120.67 (15)
C5—C6—H6	118.7 (11)	C20—C19—H19	120.6 (11)
C7—C6—H6	120.9 (11)	C18—C19—H19	118.8 (11)
C8—C7—C6	118.11 (15)	C19—C20—C21	120.16 (15)
C8—C7—H7	122.1 (12)	C19—C20—H20	121.9 (13)
C6—C7—H7	119.8 (12)	C21—C20—H20	117.9 (13)
F1—C8—C7	118.48 (16)	O4—C21—C22	124.66 (14)
F1—C8—C9	118.13 (17)	O4—C21—C20	115.13 (14)
C7—C8—C9	123.39 (16)	C22—C21—C20	120.20 (14)
C8—C9—C10	117.76 (17)	C21—C22—C23	119.12 (14)
C8—C9—H9	119.7 (13)	C21—C22—H22	120.7 (11)
C10—C9—H9	122.5 (13)	C23—C22—H22	120.2 (11)
C9—C10—C5	120.96 (15)	C18—C23—C22	121.34 (14)
C9—C10—H10	119.1 (12)	C18—C23—H23	120.2 (11)
C5—C10—H10	119.9 (12)	C22—C23—H23	118.4 (11)
O1—C11—O2	122.00 (13)	O4—C24—H24A	110.4 (13)
O1—C11—C2	127.18 (14)	O4—C24—H24B	110.9 (13)
O2—C11—C2	110.81 (11)	H24A—C24—H24B	110.1 (18)
O2—C12—C13	106.88 (13)	O4—C24—H24C	104.9 (13)
O2—C12—H12A	108.3 (12)	H24A—C24—H24C	111.1 (18)
C13—C12—H12A	112.1 (12)	H24B—C24—H24C	109.4 (18)
O2—C12—H12B	108.5 (11)	H5A—O5—H5B	104.0
			
C15—N3—N4—C17	173.45 (12)	C1—C5—C10—C9	−179.13 (15)
C4—N2—C1—C2	−21.62 (17)	C12—O2—C11—O1	1.5 (2)
C15—N2—C1—C2	167.22 (11)	C12—O2—C11—C2	−179.00 (12)
C4—N2—C1—C5	101.08 (14)	C3—C2—C11—O1	2.3 (2)
C15—N2—C1—C5	−70.08 (15)	C1—C2—C11—O1	−176.78 (15)
N2—C1—C2—C3	20.41 (18)	C3—C2—C11—O2	−177.21 (13)
C5—C1—C2—C3	−100.69 (15)	C1—C2—C11—O2	3.75 (18)
N2—C1—C2—C11	−160.54 (12)	C11—O2—C12—C13	173.66 (14)
C5—C1—C2—C11	78.36 (15)	N4—N3—C15—N2	178.26 (11)
C11—C2—C3—N1	173.50 (13)	N4—N3—C15—C16	−2.1 (2)
C1—C2—C3—N1	−7.5 (2)	C4—N2—C15—N3	177.79 (12)
C11—C2—C3—C14	−5.0 (2)	C1—N2—C15—N3	−10.60 (19)
C1—C2—C3—C14	174.03 (14)	C4—N2—C15—C16	−1.93 (17)
C4—N1—C3—C2	−7.2 (2)	C1—N2—C15—C16	169.67 (12)
C4—N1—C3—C14	171.48 (13)	N3—C15—C16—S1	−176.70 (12)
C3—N1—C4—N2	6.2 (2)	N2—C15—C16—S1	2.98 (14)
C3—N1—C4—S1	−171.99 (10)	C4—S1—C16—C15	−2.61 (11)
C15—N2—C4—N1	−178.53 (13)	N3—N4—C17—O3	−7.08 (19)
C1—N2—C4—N1	9.9 (2)	N3—N4—C17—C18	174.20 (11)
C15—N2—C4—S1	−0.18 (15)	O3—C17—C18—C23	−160.50 (14)
C1—N2—C4—S1	−171.74 (10)	N4—C17—C18—C23	18.2 (2)
C16—S1—C4—N1	−179.84 (12)	O3—C17—C18—C19	19.1 (2)
C16—S1—C4—N2	1.73 (11)	N4—C17—C18—C19	−162.14 (13)
N2—C1—C5—C10	−58.95 (16)	C23—C18—C19—C20	−1.5 (2)
C2—C1—C5—C10	60.97 (17)	C17—C18—C19—C20	178.83 (14)
N2—C1—C5—C6	121.37 (13)	C18—C19—C20—C21	1.3 (2)
C2—C1—C5—C6	−118.72 (14)	C24—O4—C21—C22	1.3 (2)
C10—C5—C6—C7	−1.9 (2)	C24—O4—C21—C20	−178.54 (16)
C1—C5—C6—C7	177.79 (13)	C19—C20—C21—O4	179.23 (14)
C5—C6—C7—C8	1.4 (2)	C19—C20—C21—C22	−0.6 (2)
C6—C7—C8—F1	179.90 (15)	O4—C21—C22—C23	−179.73 (14)
C6—C7—C8—C9	0.4 (3)	C20—C21—C22—C23	0.1 (2)
F1—C8—C9—C10	178.81 (16)	C19—C18—C23—C22	1.0 (2)
C7—C8—C9—C10	−1.7 (3)	C17—C18—C23—C22	−179.37 (13)
C8—C9—C10—C5	1.2 (3)	C21—C22—C23—C18	−0.3 (2)
C6—C5—C10—C9	0.6 (2)		

### Hydrogen-bond geometry (Å, º)

**Table:**

*D*—H···*A*	*D*—H	H···*A*	*D*···*A*	*D*—H···*A*
N4—H4···N1^i^	0.885 (19)	2.164 (19)	2.9888 (17)	154.8 (16)
C1—H1···O5^ii^	0.987 (17)	2.345 (18)	3.307 (5)	164.7 (13)
C7—H7···O4^iii^	0.97 (2)	2.41 (2)	3.285 (2)	148.7 (17)
C13—H13*B*···F1^ii^	0.98 (3)	2.49 (3)	3.386 (2)	153.0 (19)
C16—H16*A*···N1^i^	0.98 (2)	2.58 (2)	3.4019 (19)	142.4 (15)
C16—H16*B*···O5^iv^	0.98 (2)	2.37 (2)	3.282 (5)	154.6 (16)
C24—H24*A*···O1^v^	0.99 (2)	2.53 (2)	3.450 (3)	154.6 (18)
C24—H24*C*···O1^vi^	0.95 (2)	2.57 (2)	3.504 (2)	167.8 (17)
O5—H5*A*···F1	0.87	1.76	2.479 (5)	138
O5—H5*B*···O3^vii^	0.87	2.00	2.863 (5)	174

Symmetry codes: (i) −*x*+1, *y*+1/2, −*z*+1/2; (ii) *x*, −*y*+3/2, *z*+1/2; (iii) −*x*+1, −*y*+2, −*z*+1; (iv) −*x*+1, *y*−1/2, −*z*+1/2; (v) −*x*+1, −*y*+1, −*z*+1; (vi) *x*−1, *y*+1, *z*; (vii) *x*, −*y*+3/2, *z*−1/2.
